# Handover of the Editor in Chief positions – from the new Editors-in-Chief to the readership of *Respiratory Research*

**DOI:** 10.1186/s12931-019-0989-y

**Published:** 2019-02-19

**Authors:** Kelan Tantisira, Oliver Schildgen

**Affiliations:** 1 Brigham and Women’s Hospital and Harvard Medical School, Boston, MA USA; 2 Kliniken der Stadt Köln gGmbH, Klinikum der Privaten Universität Witten Herdecke, Ostmerheimer Str. 200, D-51109 Cologne, Germany

*Respiratory Research* was first published in April 2000, accompanied by an editorial written by Dr. Peter Barnes, entitled “*Respiratory Research*: a new multidisciplinary journal for a new age (http://respiratory-research.com). “It was the first respiratory medicine journal in which the online version was the primary place of publication. According to the Directory of Open Access Journals (www.doaj.org), there are now 48 open access journals in the field of respiratory medicine; among these, *Respiratory Research* has continuously retained the top InCites Journal Impact Factor. It is in this spirit that we congratulate and commend Drs. Jan Lotvall and Rey Panettieri for their dedicated and tireless service as the outgoing Editors-in-Chief. We sincerely appreciate their guidance during our recent transition period and their journal leadership over the past 10 years. Our goal is to learn from their successes and strive to continuously improve the journal in ways that directly and positively impact our readers.

At its inception, *Respiratory Research* was conceived with co-Editors-in-Chief (starting with Drs. Barnes and Jeffrey Drazen) and this continues with our tenure. This model allows for facilitation of broader ideas, enhanced efficiency in peer review, and increased breadth of knowledge in the understanding of our product. As a brief background, Oliver Schildgen is based in Cologne at the Hospital of the City of Cologne and the Private University of Witten/Herdecke, Germany’s oldest Private University. He is a recognized Ph.D. virologist and molecular pathologist focused on the pathogenesis of human respiratory viruses, including human metapneumovirus, human bocavirus, and lung cancer. Kelan Tantisira is based in Boston at the Brigham and Women’s Hospital and Harvard Medical School and is a pulmonologist known for his work in asthma epidemiology, genetics, genomics, and pharmacogenomics. Thus, our backgrounds are highly complementary, with our expertise further enhanced by our outstanding associate editors and editorial board.

Despite our diverse backgrounds, we share a common vision for *Respiratory Research*. Our first goal is to maintain the journal as the pre-eminent Open Access publication focused on advancing the understandings of lung health and disease, as established by our predecessors. We are committed to continuing to solicit and review a wide range of manuscripts spanning the breadth of respiratory medicine. We will augment this with efficient editorial review complemented by journalistic rigor and integrity. Second, we both agree that it is important to attract readership and investment by the young scientists of today, which are the stakeholders and senior authors of tomorrow. This emphasis on young readers can be further extended to early physician-scientists.

Next, we plan to begin a regular feature focusing on emerging technologies and their application to respiratory research and disease. These are areas of great evolving interest, but are currently under-represented in the *Respiratory Research* portfolio. Along a similar vein, we plan to actively pursue the opportunity to publish articles on emerging lung therapeutics, including early clinical trial results.

Finally, we remain very open and receptive to your feedback on the format and content of *Respiratory Research* and welcome your suggestions for future commentaries, reviews and other content. Please do not hesitate to contact either of us with your thoughts.

